# Risco de Morte em Idosos com Sonolência Excessiva Diurna, Insônia e Depressão: Estudo de Coorte Prospectiva em População Urbana no Nordeste Brasileiro

**DOI:** 10.36660/abc.20200059

**Published:** 2021-06-10

**Authors:** Johnnatas Mikael Lopes, Fábio Dantas Galvão, Angelo Giuseppe Roncalli da Costa Oliveira

**Affiliations:** 1 Universidade Federal do Vale do São Francisco Paulo Afonso BA Brasil Universidade Federal do Vale do São Francisco (UNIVASF) - Colegiado de Medicina, Paulo Afonso, BA - Brasil; 2 Universidade Estadual da Paraíba Campina Grande PB Brasil Universidade Estadual da Paraíba - Fisioterapia, Campina Grande, PB - Brasil; 3 Universidade Federal do Rio Grande do Norte Natal RN Brasil Universidade Federal do Rio Grande do Norte - Odontologia, Natal, RN - Brasil

**Keywords:** Transtornos do Sono-Vigília/complicações, Idoso, Disfunções Psicológicas, Mortalidade, Depressão

## Abstract

**Fundamento:**

A íntima relação entre a regulação do sono e os eventos cardiovasculares é um dos principais focos de investigação na medicina contemporânea. Hábitos e características do sono interferem na ritmicidade cardíaca e também na expectativa de vida, principalmente em idosos.

**Objetivo:**

Estimar o risco de óbito e de eventos cardiovasculares em idosos comunitários que apresentam queixa de insônia e sonolência excessiva diurna ao longo de oito anos de seguimento.

**Método:**

Foi desenhada uma coorte prospectiva com 160 idosos, a primeira onda em 2009 e a segunda em 2017. Os grupos de seguimento foram determinados pela exposição ou não às queixas de insônia primária e a sonolência excessiva diurna, com ou sem ronco. As covariáveis sexo, estado conjugal, depressão, hipertensão e diabetes foram controladas. O desfecho primário foi o óbito e o secundário, os eventos cardiocerebrovasculares (ECV). As probabilidades dos desfechos foram estimadas pelo risco relativo (RR), através da regressão de Poisson, adotando-se α ≤ 0,05.

**Resultados:**

Registraram-se 40 mortes no período (25,97%:19,04-32,89) e 48 ECVs (30,76%:23,52-38,01). Os homens apresentaram maior risco (RR = 1,88;1,01-3,50) de óbito. A depressão (RR = 2,04;1,06-3,89), a gravidade da insônia (RR = 2,39;1,52-4,56) e a latência do sono entre 16-30 minutos (RR = 3,54;1,26-9,94) e 31-60 minutos (RR = 2,23;1,12-4,47) aumentaram independentemente o risco de óbito em idosos comunitários. Os ECVs foram preditos apenas por idosos hipertensos e/ou diabéticos (RR = 8,30; 1,98-34,82).

**Conclusão:**

A mortalidade em idosos é influenciada pelo estado emocional e pela dificuldade de dormir, diferentemente dos ECVs, condicionados apenas pelas condições pressóricas arteriais e metabólicas.

## Introdução

Os transtornos do sono parecem estar relacionados, de modo independente, com desfechos graves de saúde como os eventos cardiovasculares^1 , 2^ e o óbito.^3 - 5^ Destacam-se a insônia e os sintomas de sonolência excessiva diurna (SED), condições do sono possivelmente associadas a alterações neurofisiológicas^6^ e psicogênicas.^7^

Existem evidências consistentes que apontam para a relação da desregulação do ciclo sono-vigília e das funções cognitivas com processos inflamatórios cerebrais,^8^ assim como déficit no automatismo circulatório,^9^ o que produz sobrecarga cardíaca. As queixas relacionadas ao sono são muito comuns em pacientes com morbidades cardíacas, neurológicas e psicológicas. As principais delas, insônia e SED, são manifestações clínicas comumente mais brandas e podem surgir previamente ou em estágios iniciais de transtornos do sono mais debilitantes como a apneia obstrutiva do sono.^10^

Estudos brasileiros recentes revelaram a associação entre risco cardiovascular, insônia e SED.^11^ Em outros contextos, Lee et al.^12^ evidenciaram, em uma coorte de pacientes coronários, que a presença de SED era um fator preditivo em eventos cardiovasculares futuros, quando controlados outros fatores intervenientes.^12^ No entanto, é difícil saber se a SED surgiu antes ou depois das coronariopatias.

A insônia também está relacionada com os eventos cardiovasculares. Assim como a SED, ela se revela um potencial fator de risco para eventos cerebrovasculares.^2^ Achados apontam que a insônia está associada à mortalidade geral apenas em indivíduos do sexo masculino, mas se diferencia de acordo com os contextos.^13^

Além dessas condições clínicas, associadas ou independentes, vários parâmetros relacionados com o sono podem estar influenciando as mortes e os eventos cardiovasculares como a eficiência do sono, duração do sono, uso de medicamentos para dormir e movimentos involuntários do corpo.^13^ Acrescenta-se ainda a interação com fatores não modificáveis como o gênero, e modificáveis como as características comportamentais, estilo de vida e fatores emocionais. Podemos citar também os fatores sociais como a escolaridade e o estrato social, que sugerem relação com alterações no ritmo biológico do sono.^14^

Todavia, são incipientes as evidências dessas relações na população brasileira, principalmente em subgrupos vulneráveis em níveis biológico e social como os idosos, cuja proporção na população cresce em ritmo constante, mesmo em regiões com características acentuadas de subdesenvolvimento, como a região Nordeste.

Dessa forma, objetiva-se investigar como a presença de queixas de insônia e/ou SED, assim como parâmetros da qualidade do sono e sintomas depressivos influenciam a longo prazo na mortalidade e em eventos cardiovasculares nos indivíduos idosos comunitários. Conjecturamos a hipótese de que a presença dessas dissonias, isolada ou em conjunto com sintomas depressivos, modulam a probabilidade de óbito e eventos cardiovasculares em idosos.

## Método

### Desenho

Desenvolveu-se um estudo de coorte prospectiva, no qual a população de base para a pesquisa se constituiu de indivíduos idosos e residentes na zona urbana da cidade de Campina Grande, no estado da Paraíba. A amostra foi de idosos participantes de pesquisa transversal em 2009, desenvolvida por Lopes, Dantas e Medeiros^11^ na mesma cidade, sendo considerada a primeira onda de coleta de dados com 160 participantes, dimensionados para um estudo de prevalência de SED e insônia. A segunda onda de coleta de dados ocorreu no ano de 2017, para estimar a ocorrência dos desfechos de óbito e eventos cardiovasculares.

O dimensionamento amostral para estimar o risco de óbito no grupo de exposição (com SED e/ou insônia) com relação ao grupo que não foi exposto considerou um desfecho estimado de 10% neste grupo e de 30% no grupo sob exposição,^10^ junto a um nível de confiança de 95% para minimizar um erro tipo I e um poder de teste de 80% para minimizar a ocorrência de erro tipo II. Dessa forma, mostrou-se necessário um mínimo de 48 participantes em cada grupo para que o seguimento pudesse estimar os desfechos de modo adequado. De toda a forma, identificaram-se na primeira onda 114 idosos como pertencentes ao grupo de expostos.

Justifica-se considerar a exposição SED e a insônia conjunta em virtude da frequente e concomitante ocorrência de ambas na amostra investigada, além de serem eventos simbióticos em idosos.

Incluíram-se na amostra indivíduos de ambos os gêneros com idade acima de 60 anos, rastreados na primeira onda no ano de 2009 e que se disponibilizaram a participar do estudo. Os indivíduos com incapazes de responder aos questionamentos e de serem avaliados fisicamente foram excluídos da pesquisa.

A escolha dos participantes na primeira onda foi aleatória dentro dos 49 bairros do perímetro urbano estabelecido pela Prefeitura da Cidade de Campina Grande (PB). As ruas sorteadas foram percorridas de uma extremidade a outra, nas duas laterais, saltando-se nove casas a partir da esquina escolhida como início, de acordo com o método utilizado pelo Instituto Brasileiro de Geografia e Estatística (IBGE) para aleatorização neste município. Essa alternância era dada pela razão entre o número total de domicílios e o número de idosos a serem visitados no bairro. Caso não houvesse idoso no domicílio selecionado, a busca deveria ser feita na residência mais próxima. Quando havia mais de um idoso no local, a coleta de dados era feita com todos eles.

### Variáveis e instrumentos de coleta de dados

Os participantes responderam, na primeira onda, ao formulário sociodemográfico com dados sobre sexo (homem ou mulher), idade (em anos), escolaridade (analfabeto, ensino fundamental, ensino médio ou ensino superior) e situação conjugal (com ou sem companheiro). Além disso, algumas informações sobre a existência de condição de saúde crônica, como hipertensão e diabetes, foram averiguadas para efeito de controle de confusão, por serem as doenças crônicas mais prevalentes em idosos. Identificou-se de modo dicotômico o diagnóstico de hipertensão e/ou diabetes nos prontuários das unidades de saúde, assim como a inclusão no programa Hiperdia. Todas as variáveis da primeira onda constituíram-se em independentes na coorte.

Para avaliar a presença de sonolência excessiva diurna, utilizou-se a Escala de Sonolência de Epworth ou Escala de Cansaço.^15^ Identificaram-se com SED aqueles com pontuação acima de 10 na Escala de Cansaço, sendo o grau leve entre 11 e 16 e acima deste, o último escore e grau grave. As queixas de insônia foram avaliadas de modo subjetivo a partir de um interrogatório a respeito da dificuldade de iniciar o sono, de manter-se no estado de sono e da dificuldade de retornar ao sono sem motivo aparente.^15^ Assim, criou-se um indicador composto que avaliava a presença de SED e/ou insônia, chamado Indicador de Transtorno do Sono.

A latência para o sono foi determinada a partir de domínios do Índice de Qualidade do Sono de Pittsburgh (IQSP), que avalia a qualidade do sono em sete domínios: qualidade subjetiva, latência do sono, duração do sono, eficiência do sono, transtornos do sono, uso de medicação para dormir e disfunção durante o dia. A pontuação varia de 0 a 20. O sono de qualidade ruim é caracterizado em indivíduos com pontuação maior que 5. O ronco foi estimado pela Escala de Ronco de Stanford.^15^

Em todos os participantes foi medido o índice de massa corpórea (IMC), que é baseado na massa e altura do indivíduo. O peso era obtido com balança da marca GEOM^®^, modelo B8030, com limite de peso de 150 kg e sensibilidade de 100 g. A altura foi mensurada pelo estadiômetro compacto adulto do tipo trena, da marca Wiso^®^, graduado em centímetros. Quando o participante não apresentava condições de caminhar até a balança ou de adotar a postura ortostática para medição da altura, era excluído da pesquisa. Além disso, foram coletados dados de circunferência abdominal (CA) com fita métrica inelástica, classificando os indivíduos em participantes de baixo e alto risco cardiovascular e estabelecendo como ponto de corte as seguintes referências: < 84 cm para mulheres e < 104 cm para homens.^16^ Construiu-se um índice nutricional dicotômico considerado adequado (IMC < 27 kg/m^2^ e/ou de baixo risco cardiovascular) e inadequado (IMC ≥ 27 Kg/m2 e/ou de alto risco cardiovascular).

Para avaliar a existência de sintomas depressivos no idoso, utilizou-se a Escala de Depressão Geriátrica de Sheikh e Yesavage,^17^ em que as pontuações de 0 a 10 equivalem ao estado normal; de 11 a 20, remetem aos quadros depressivos médios; e de 21 a 30, indicam possível depressão moderada/grave.

Os pesquisadores foram devidamente treinados para utilizarem os instrumentos de coleta de dados de modo confiável antes de aplicarem o questionário socioeconômico, a antropometria, a Escala Geriátrica de Depressão e a Escala de Sonolência de Epworth e do IQSP.

A segunda etapa da pesquisa foi realizada em 2017. Inicialmente, os pesquisadores utilizaram a estratégia do contato telefônico, seguida da visita domiciliar, visita à Unidade Básica de Saúde e contato com informantes-chave (vizinhos e parentes) para a localização dos participantes e desfechos. Após a localização, para estimar os desfechos, era constatado o estado vital (vivo ou morto) do participante e a ocorrência de disfunções cardiovasculares e cerebrovasculares. Os eventos de infarto agudo do miocárdio, arritmias, valvulopatias, ataque isquêmico transitório e acidente vascular cerebral isquêmico ou hemorrágico, entre outros, foram autorrelatados pelos participantes ou seus familiares, e confirmados e/ou acrescentados a partir dos prontuários nas unidades de saúde e ou atestados de óbito para os indivíduos já falecidos.

### Análise de dados

A análise inferencial foi realizada com o desfecho primário “óbito” e o secundário “eventos cardiocerebrovasculares”, controlando o efeito de covariáveis como hipertensão e diabetes. Estimou-se o risco relativo para as variáveis independentes de agrupamento SED/insônia, bem como os sintomas depressivos, os parâmetros da qualidade do sono e obesidade a partir da Modelagem Linear Generalizada, com modelo baseada na distribuição de Poisson e função ligante logarítmica linear. Diante dessa situação inferencial, adotou-se um nível de significância de 5% para minimizar um erro tipo I. Foi utilizado o *software* livre R para a análise descritivas e inferenciais de dados.

A pesquisa foi submetida ao Comitê de Ética em Pesquisa do Hospital Universitário Onofre Lopes, em respeito à Resolução n^o^ 466/2012 do Conselho Nacional de Saúde, obtendo o parecer 2.048.708.

## Resultados

Foram acompanhados 160 idosos, com idade entre 60 a 98 anos, média de 72,16 ± 7,84 anos, sendo 112 mulheres (71,2%) e 135 indivíduos cuja escolaridade máxima era o ensino fundamental completo (84,4%). O tempo médio de acompanhamento foi de 5,3 anos, com apenas quatro perdas de seguimento.

Nesta amostra, 118 indivíduos eram normais quanto à SED (73,75%:66,93-80,56), 30 indivíduos apresentavam grau leve de SED (18,75%:12,70-24,79) e 12 indivíduos se encontravam com a forma grave de SED (7,50%:3,41-11,58). A insônia esteve presente em 98 idosos (54,9%), sendo que 65 indivíduos a apresentavam no estágio moderado/grave (43,0%). Registrou-se o desfecho “morte” em 40 idosos (25,97%:19,04-32,89) e o desfecho de evento cardiovascular aconteceu em 48 indivíduos (30,76%:23,52-38,01). A Tabela 1 descreve a distribuição das outras variáveis independentes, bem como sua modelagem preditiva.

**Tabela 1:** – Análise descritiva, Modelo bruto e ajustado para o risco de óbito em idosos do município de Campina Grande, Paraíba, Brasil, 2009-2017

	Risco de morte				
Variáveis	Modelo bruto			Modelo ajustado	
	n (%)	RR	IC 95%	RR	IC 95%
**Sexo**					
Homem	44 (28,2)	1,69	1,01-2,87	1,73	0,92-3,28
Mulher	112 (71,8)	1	-	1	-
**Situação conjugal**					
Com companheiro(a)	37 (23,7)	1,59	0,91-2,77	1,50	0,86-2,62
Sem companheiro(a)	119 (76,3)	1	-	1	-
**Depressão**					
Sim	66 (42,3)	1,66	0,97-2,84	2,39	1,52-4,56
Não	90 (57,7)	1	-	1	-
**Índice nutricional**					
Inadequado	36 (23,1)	0,76	0,42-1,38	1,12	0,58-2,18
Adequado	120 (76,9)	1	-	1	-
**Hipertensão/diabetes**					
Sim	114 (73,1)	0,86	0,48-1,53	0,84	0,46-1,51
Não	42 (26,9)	1	-	1	-
**Indicador de transtorno do sono**					
Insônia/SED	114 (81,3)	0,91	0,40-2,07	0,62	0,33-1,16
Normal	46 (28,7)	1	-	1	-
**IQSP**					
Bom	62 (45,9)	0,78	0,43-1,41	0,62	0,33-1,16
Ruim	73 (54,1)	1	-	1	-

*IC 95%: intervalo de confiança de 95%; RR: risco relativo; SED: sonolência excessiva diurna; IQSP: Índice de Qualidade do Sono de Pittsburg.*

A modelagem de Poisson revelou, após ajustamento, que entre as variáveis independentes estudadas, apenas a presença de sintomas depressivos é considerada fator de risco para óbito em idosos comunitários. Os idosos com depressão apresentavam 2,39 vezes mais possibilidades de falecer do que os não mostravam sintomas depressivos (IC 95%:1,52-4,56). Por outro lado, os indicadores compostos de estado nutricional, os transtornos do sono, a qualidade do sono e até mesmo a hipertensão/diabetes não foram capazes de influenciar na taxa de mortalidade durante o acompanhamento de oito anos do estudo ( Tabela 2 ).

**Tabela 2:** – Modelo ajustado segregado em morbidades específicas do sono para o risco de óbito em idosos do município de Campina Grande, Paraíba, Brasil, 2009-2017

Variáveis	Risco de morte		
		Modelo ajustado	
	n (%)	RR	IC 95%
**Sexo**			
Homem	44 (28,2)	1,88	1,01-3,50
Mulher	112 (71,8)	1	-
**Situação conjugal**			
Com companheiro(a)	37 (23,7)	2,10	1,20-3,68
Sem companheiro(a)	119 (76,3)	1	-
**Depressão**			
Sim	66 (42,3)	2,04	1,06-3,89
Não	90 (57,7)	1	-
**Hipertensão/diabetes**			
Sim	114 (73,1)	1,19	0,54-2,60
Não	42 (26,9)	1	-
**Índice nutricional**			
Inadequado	36 (23,1)	1,27	0,59-2,71
Adequado	120 (76,9)	1	-
**Latência do sono**			
Mais 60 min	22 (16,2)	1,68	0,74-3,81
Entre 31-60 min	19 (14,0)	3,54	1,26-9,94
Entre 16-30 min	31 (22,8)	2,23	1,12-4,47
Menos de 15 min	64 (47,1)	1	-
**Gravidade da insônia**			
Moderada/grave	65 (43,0)	0,94	0,29-2,96
Leve	33 (21,9)	2,30	1,08-4,89
Sem insônia	53 (35,1)	1	-
**Gravidade do ronco**			
Ronco excessivo	38 (26,2)	0,69	0,31-1,53
Ronco leve	54 (37,2)	1,24	0,58-2,65
Sem ronco	53 (36,6)	1	-
**SED**			
Moderada/grave	12 (7,7)	1,08	0,43-2,68
Leve	29 (18,6)	0,60	0,24-1,53
Sem SED	115 (73,7)	1	-

*IC95%: intervalo de confiança de 95%; RR: risco relativo; SED: sonolência excessiva diurna.*

Ao analisar as variáveis constituintes dos indicadores compostos inclusos no modelo anterior, identifica-se uma nova distribuição para o desfecho “óbito” ( Tabela 3 ) e apresentar sintomas depressivos continuou sendo um fator de risco. Todavia, percebe-se que existe uma predição maior de óbito para o sexo masculino, 88% maior que nas mulheres (RR = 1,88;1,01-3,50) e também nos idosos com companheiro(a) (RR = 2,10; 1,20-3,68). De modo semelhante, os indicadores de latência de sono entre 16-30 minutos (RR = 2,23;1,12-4,47) e entre 31-60 minutos (RR = 3,54;1,26-9,94) predizem mais óbitos que nos indivíduos que dormem antes de 15 minutos. Apresentar insônia leve também foi considerado preditor de óbito nesta amostra; os idosos que apresentam grau leve possuem 2,30 vezes mais possibilidades de falecer (IC 95%:1,08-4,89).

**Tabela 3:** – Modelo preditivo para o risco de evento cardiocerebrovascular em idosos do município de Campina Grande, Paraíba, Brasil, 2009-2017

Variáveis	Risco de ECC			
	Modelo bruto		Modelo ajustado	
	RR	IC 95%	RR	IC 95%
**Sexo**				
Homem	1,19	0,72-1,98	1,23	0,69-2,21
Mulher	1	-	1	-
**Situação conjugal**				
Com companheiro(a)	0,98	0,55-1,72	1,03	0,58-1,82
Sem companheiro(a)	1	-	1	-
**Depressão**				
Sim	1,14	0,71-1,83	1,29	0,78-2,15
Não	1	-	1	-
**Índice nutricional**				
Inadequado	0,64	0,33-1,25	0,94	0,55-1,62
Adequado	1	-	1	-
**Hipertensão/diabetes**				
Sim	4,43	1,69-11,64	5,72	1,87-17,46
Não	1	-	1	-
**Indicador de transtorno do sono**				
Sim	0,72	0,32-1,63	0,66	0,31-1,38
Não	1	-	1	-
IQSP				
Bom	1,27	0,76-2,10	1,36	0,82-2,27
Ruim	1	-	1	-

*IC 95%: intervalo de confiança de 95%; RR: risco relativo; SED: sonolência excessiva diurna; IQSP: Índice de Qualidade do Sono de Pittsburg.*

No desfecho de evento cardiocerebrovascular durante o acompanhamento de oito anos, a única variável independente que demonstrou capacidade preditiva foi o indicador de hipertensão/diabetes. Idosos hipertensos e/ou diabéticos apresentavam probabilidade quase seis vezes maior (RR = 5,72; IC 95%:1,87-17,46) de ter disfunções cardiovasculares e cerebrais que os idosos normotensos e com metabolismo glicérico normal. Os indicadores compostos e segregados do estado nutricional, o transtorno do sono e a qualidade do sono não revelam nenhum efeito no período analisado ( Tabela 4 ).

**Tabela 4:** – Modelo preditivo segregado em morbidades específicas do sono para o risco de eventos cardiocerebrovasculares em idosos do município de Campina Grande, Paraíba, Brasil, 2009-2017

Variáveis	Risco de morte	
	Modelo ajustado	
	RR	IC 95%
**Sexo**		
Homem	1,38	0,59-3,21
Mulher	1	-
**Situação conjugal**		
Com companheiro(a)	1,32	0,62-2,82
Sem companheiro(a)	1	-
**Depressão**		
Sim	1,32	0,67-2,59
Não	1	-
**Hipertensão/diabetes**		
Sim	8,30	1,98-34,82
Não	1	-
**Índice nutricional**		
Inadequado	1,18	0,58-2,41
Adequado	1	-
**Latência de sono**		
Mais 60 min	1,03	0,40-2,62
Entre 31-60 min	1,34	0,50-3,55
Entre 16-30 min	1,09	0,45-2,59
Menos de 15 min	1	-
**Gravidade da insônia**		
Moderada/grave	1,35	0,55-3,33
Leve	2,14	0,84-5,42
Sem insônia	1	-
**Gravidade do ronco**		
Ronco excessivo	0,99	0,41-2,41
Ronco leve	0,96	0,44-2,07
Sem ronco	1	-
**SED**		
Moderada/grave	0,79	0,26-2,43
Leve	0,74	0,28-1,97
Sem SED	1	-

*IC 95%: intervalo de confiança de 95%; RR: risco relativo; SED: sonolência excessiva diurna.*

## Discussão

Evidencia-se no presente estudo que os desfechos de óbito e eventos cardiovasculares estudados são preditos por exposições distintas. O falecimento de idosos comunitários é fortemente influenciado pela ocorrência de sintomas depressivos, sendo mais comum em homens e nos indivíduos que vivem com companheiro(a). Entre as características do sono, destaca-se a elevada latência para iniciar o sono e a gravidade da insônia como fatores de risco para o óbito em idosos. Por outro lado, apenas os idosos hipertensos e/ou diabéticos apresentaram mais riscos de desenvolver desfechos cardiovasculares.

Diversos estudos têm esclarecido sobre o maior risco de óbito entre homens, principalmente por condições crônicas como as doenças cardiovasculares em idosos.^18^ O *status* conjugal também é identificado como condição relacionada com a vulnerabilidade de saúde. De modo geral, indivíduos sem companheiros apresentam menores suportes sociais e maior predisposição aos eventos prejudiciais na saúde, como o óbito.^19^ Todavia, nossos achados sugerem que idosos com companheiro(a) são mais vulneráveis. A hipótese explicativa aventada está alicerçada nas características da população estudada, em que a possibilidade de interação entre baixas condições socioeconômicas e baixo apoio social sobrecarregam os cônjuges no cuidado recíproco ou de um indivíduo vulnerável. Dados da Pesquisa Nacional de Saúde apontam também uma maior ocorrência de declínio na reparação do sono em indivíduos com companheiro(a) no Brasil.

Evidenciou-se que sintomas depressivos em idosos são fatores de risco para óbito. Os sintomas depressivos prediziam eventos desta natureza para todas as causas, apresentando interação com o sexo masculino.^20^ Identificou-se também que os sintomas depressivos são preditivos na mortalidade por isquemia cardíaca em uma coorte da população geral na Inglaterra,^21^ o que foi corroborado com evidências que indicam a depressão como a principal morbidade crônica que atualmente atinge os idosos^22^ e que pode ter efeito direto ou interação com outros mecanismos patológicos.

De modo geral, quadros depressivos estão relacionados com as alterações do sono. Lima at al.^23^ relataram que idosas com menor tempo de sono apresentam piores indicadores de saúde mental nos aspectos emocionais em estudo populacional no Brasil.^23^ Sintomas de insônia e sonolência são preditivos para depressão, e é possível que estas condições estabeleçam relação de retroalimentação, que culminam no desfecho de óbito.^3^

A depressão como fator preditivo de mortalidade em idosos está relacionada com a potencialização de comorbidades crônicas.^24^ A depressão em idosos, quando associada a morbidades crônicas, dificulta o manejo das outras condições, como diabetes e hipertensão, principalmente com relação à aderência ao tratamento e ao autocuidado.^25^ Acrescenta-se também à modificação da percepção da dor^26^ e do estado geral de saúde,^4^ o que cria manifestações clínicas superdimensionadas ao real problema fisiopatológico.

Nossos achados apontam para uma exposição de risco de óbito produzida pela latência do sono aumentada e a gravidade da insônia em idosos. De modo semelhante, Lallukka et al.^3^ revelaram que na Noruega e Finlândia a insônia aumenta a probabilidade de óbito em homens,^3^ assim como nos Estados Unidos,^27^ sobretudo a dificuldade de iniciar o sono. A insônia está fortemente associada a outros fatores que predizem óbito, como a duração do sono reduzida ou elevada^28^ e a depressão em idosos. Por outro lado, Chen et al.^29^ Identificaram, em nove anos de segmento, independência entre insônia e eventos de óbito em idosos asiáticos.^29^ Tais discrepâncias podem advir de diferenças contextuais ou de tempo de seguimento insuficiente para a ocorrência dos desfechos.

Diferentemente de outros estudos, o indicador composto de queixas de sonolência excessiva diurna e insônia, bem como seus indicadores simples, não se mostram fatores preditivos para eventos cardiovasculares. Todavia, Wu et al.^2^ evidenciaram por meio de coorte em Taiwan que a insônia aumenta o risco de evento cerebrovascular, principalmente em adultos jovens se comparados a idosos. Eventos cardíacos também foram preditos, pela presença de sintomas de insônia em outros achados epidemiológicos.^10 , 30^

Para o desfecho de eventos cardiovasculares, nossos dados não apontam as condições do sono ou emocionais como fatores de risco. O único preditor de risco identificado foi a condição de saúde de hipertensão e/ou diabetes nos idosos durante o período analisado. A literatura científica já evidencia, com bastante respaldo, os efeitos deletérios que a hipertensão arterial sistêmica e o diabetes produzem na sociedade contemporânea, sendo um robusto fator de risco ou componente para eventos cerebrovasculares isquêmicos^31^ e hemorrágicos, isquemia cardíaca e síndrome metabólica.^32^

Alguns pontos merecem destaque na interpretação dos nossos achados. Apesar do rigor metodológico no planejamento do seguimento, não foram controladas variáveis sociais importantes como renda, rede de suporte social ou status social que refletiria satisfatoriamente aspectos contextuais implicantes nas exposições e desfechos. A baixa escolaridade da população estudada não permitiu uma boa variabilidade desta característica e, portanto, maiores inferências sobre seus efeitos. A falta de bom conhecimento da saúde e/ou acesso aos serviços de saúde da amostra impediu a identificação mais precisa de possíveis eventos cardiovasculares subclínicos. Sua identificação foi realizada por autorrelato. A inexistência de exame diagnóstico da apneia do sono tornou impossível controlar o efeito desta condição de saúde na sua provável interação com as características do sono. Por fim, o ajustamento de covariáveis foi limitado pelo tamanho da amostra.

Contudo, foram obtidas informações de grande valor para população idosa estudada, a qual está à margem dos grandes centros urbanos do Brasil, localizada em uma região com características peculiares quanto aos aspectos sociodemográficos, culturais e econômicos e que apresenta escassos estudos de seguimento para subpopulações vulneráveis. Dessa forma, a identificação dos efeitos independentes que o sono e os sintomas depressivos produzem sobre a taxa de mortalidade de idosos são de considerável importância nas políticas na saúde da pessoa idosa, principalmente para o planejamento público e clínico. Essas informações servirão para alicerçar as tomadas de decisão na organização do cuidado da pessoa idosa, sobretudo para mitigar os predisponentes de vulnerabilidade de sua capacidade funcional e sobrevida.

## Conclusão

Identificamos na amostra estudada efeitos independentes que os sintomas depressivos, a latência do sono para dormir e a gravidade da insônia produzem na taxa mortalidade de pessoas idosas. Tais condicionantes caracterizam-se por sua capacidade de mudança e, portanto, pela possibilidade de minimizar a perspectiva de óbito caso sejam combatidos com o manejo mais adequado e com o nível de atenção apropriado. Somam-se também os efeitos relacionados à questão de gênero masculino, que estão mais propensos à óbito. Tal fator remete a necessidade de adoção de estratégias setoriais que estejam relacionadas tanto ao acesso aos serviços de saúde como aos modos de produção de trabalho e assistência social, a fim de dirimir as inequidades.

Quanto aos eventos cardiocerebrovasculares, apenas a presença de hipertensão e/ou diabetes aumenta o seu risco em idosos comunitários. Apesar de bem conhecido como fatores de risco, existem lacunas no manejo apropriado destas condições quando se refere à adesão ao tratamento e o acesso aos serviços de saúde e seus insumos, que são influenciados por questões sociais de renda, educação e gênero, a fim de conseguir melhores resultados no âmbito da saúde pública.
