# Supplementary material for: Risco de Morte em Idosos com Sonolência Excessiva Diurna, Insônia e Depressão: Estudo de Coorte Prospectiva em População Urbana no Nordeste Brasileiro
Source: Arq Bras Cardiol. 2021 Jun 10;117(3):446–54. [Article in Portuguese] doi: 10.36660/abc.20200059 (PMC8462963; doi:10.36660/abc.20200059)
Supplement: Translation

# Translation: Risk of Death in the Elderly with Excessive Daytime Sleepiness, Insomnia and Depression: Prospective Cohort Study in an Urban Population in Northeast Brazil

**Authors:** Johnnatas Mikael Lopes, Fábio Dantas Galvão, Angelo Giuseppe Roncalli da Costa Oliveira

**Affiliations:** 1 Brazil Universidade Federal do Vale do São Francisco (UNIVASF) - Colegiado de Medicina, Paulo Afonso, BA - Brazil; 2 Brazil Universidade Estadual da Paraíba - Fisioterapia, Campina Grande, PB - Brazil; 3 Brazil Universidade Federal do Rio Grande do Norte - Odontologia, Natal, RN - Brazil

**Keywords:** Sleep Wake Disorders/complications, Aged, Psychological Dysfunction, Mortality, Depression

## Abstract

**Background:**

The close relationship between sleep regulation and cardiovascular events is one of the main focuses of research in contemporary medicine. Sleep habits and characteristics interfere with the cardiac rhythm and also with life expectancy, especially in the elderly.

**Objective:**

To estimate the risk of death and cardiovascular events in community-dwelling elderly individuals complaining of insomnia and excessive daytime sleepiness over eight years of follow-up.

**Method:**

A prospective cohort was designed with 160 elderly, with the first wave occurring in 2009 and the second in 2017. Follow-up groups were determined by exposure or not to complaints of primary insomnia and excessive daytime sleepiness with or without snoring. The covariates gender, marital status, depression, hypertension and diabetes were controlled. The primary outcome was death and the secondary outcome was cardio-cerebrovascular events (CCV). Outcome risks were estimated by relative risk (RR) through Poisson regression, adopting α≤0.05.

**Results:**

There were 40 (25.97%: 19.04-32.89) deaths over the period and 48 (30.76%: 23.52-38.01) CCV. Men had a higher risk (RR = 1.88; 1.01-3.50) of death. Depression (RR = 2.04; 1.06-3.89), insomnia severity (RR = 2.39; 1.52-4.56) and sleep latency between 16-30 minutes (RR = 3, 54; 1.26-9.94) and 31-60 minutes (RR = 2.23; 1.12-4.47) increased the risk of death independently in community-dwelling elderly. CCV were predicted only in the hypertensive and / or diabetic elderly (RR = 8.30; 1.98-34.82).

**Conclusion:**

Mortality in the elderly is influenced by the emotional state and difficulty in falling asleep, unlike CCVs, which are conditioned only by arterial and metabolic blood pressure conditions.

## Introduction

Sleep disorders seem to be independently related to serious health outcomes, such as cardiovascular events^1 , 2^ and death.^3 - 5^ Insomnia and symptoms of excessive daytime sleepiness (EDS) stand out, which are sleep conditions possibly associated with neurophysiological^6^ and psychogenic^7^ changes.

There is consistent evidence pointing to the relationship between dysregulation of the sleep-wake cycle and cognitive functions with brain inflammatory processes,^8^ as well as a deficit in circulatory automatism,^9^ resulting in cardiac overload. Complaints related to sleep are very common in patients with cardiac, neurological and psychological morbidities. The main ones, insomnia and EDS, are milder clinical manifestations and may appear previously or in the initial stages of more debilitating sleep disorders, such as obstructive sleep apnea.^10^

Recent studies in Brazil revealed an association between cardiovascular risk, insomnia and EDS.^11^ In other contexts, Lee et al.^12^ showed, in a cohort of coronary patients, that the presence of EDS was a predictive factor for future cardiovascular events when other intervening factors were controlled.^12^ However, it is difficult to know whether EDS appeared before or after coronary disease.

Insomnia is also related to cardiovascular events. Like EDS, it has shown to be a potential risk factor for cerebrovascular events.^2^ Findings indicate that insomnia is associated with general mortality in men only, although it differs according to the contexts.^13^

In addition to these clinical conditions, either in an associated or independent manner, several parameters related to sleep may influence the occurrence of death and cardiovascular events, such as sleep efficiency, sleep duration, use of sleeping medications and involuntary body movements.^13^ There is also the interaction with non-modifiable factors, such as gender and modifiable factors such as behavioral, lifestyle and emotional characteristics. Moreover, there are also social factors, such as level of schooling and social strata, which suggest a relationship with changes in the biological sleep rhythm.^14^

However, the evidence of these relationships in the Brazilian population is incipient, mainly in biologically and socially vulnerable subgroups, such as the elderly, whose proportion in the population has grown steadily even in regions with greater underdevelopment characteristics, as in the northeast region.

Thus, this article aims to investigate how the presence of insomnia and / or EDS complaints, as well as the parameters of sleep quality and depressive symptoms influence mortality and cardiovascular events in elderly community members in the long-term. We hypothesize that the presence of these dyssomnias, alone or in conjunction with depressive symptoms, modulate the likelihood of death and cardiovascular events in the elderly.

## Method

### Design

A prospective cohort study was performed. The base population for the research consists of elderly individuals living in the urban area of the city of Campina Grande, state of Paraíba, Brazil. The sample consisted of elderly participants from a cross-sectional survey carried out in 2009, developed by Lopes, Dantas and Medeiros,^11^ in the same city and which was considered the first wave of data collection with 160 participants, designed for a study on the prevalence of EDS and insomnia. The second wave of data collection took place in 2017 to estimate the occurrence of death outcomes and cardiovascular events.

The sample size to estimate the risk of death in the exposure group (with EDS and/or insomnia), in relation to the group without exposure, considered an estimated outcome of 10% in the group without exposure and 30% in the group under exposure,^10^ together with a 95% confidence interval to minimize type I error and 80% test power to minimize the occurrence of type II error. Thus, a minimum of 48 participants in each group was necessary for the follow-up to be able to adequately estimate the outcomes. In any case, 114 elderly people were identified in the first wave as belonging to the exposed group.

It is justified to consider EDS and insomnia exposure together due to the frequent concomitant occurrence of both in the investigated sample, in addition to being symbiotic events in the elderly.

The sample included individuals of both genders over the age of 60, screened in the first wave in 2009 and available to participate in the study. Individuals unable to answer questions and to be physically assessed were excluded from the sample.

The choice of participants in the first wave was randomly carried out in the 49 neighborhoods of the urban perimeter established by the city of Campina Grande-PB. The streets, selected by drawing lots, were crossed from one end to the other, on both sides, skipping nine households from the corner chosen as the starting point, according to the method used by the Brazilian Institute of Geography and Statistics (IBGE, *Instituto Brasileiro de Geografia e Estatística* ) for randomization in this municipality. This alternation was given by the ratio between the total number of households in the neighborhood and the number of elderly people to be visited in that neighborhood. If there were no elderly persons in the selected household, the nearest one was enrolled. When there was more than one elderly person in the household, data collection was carried out with all of them.

### Variables and Data Collection Instruments

The participants completed, in the first wave, the socio-demographic form containing data on gender (male or female), age (in years), level of schooling (illiterate, elementary school, high school, higher education), marital status (with or without a partner). In addition, some information about the presence of chronic health conditions, such as hypertension and diabetes, was investigated for the purpose of confounder control, as they are the most prevalent chronic diseases in the elderly. The diagnosis of hypertension and / or diabetes was dichotomously identified in the medical records of the health units, as well as the inclusion in the Hiperdia program. All variables of the first wave were independent variables in the cohort.

To assess the presence of excessive daytime sleepiness, the Epworth Sleepiness Scale (ESS) or Fatigue Scale^15^ was used. Those with a score > 10 on the ESS scale were identified with EDS; scores between 11 and 16 were considered mild cases, and above the last score were considered severe cases. Complaints of insomnia were subjectively assessed based on the answers to the questions regarding the difficulty of initiating sleep, staying asleep and difficulty returning to sleep for no apparent reason.^15^ Thus, a composite indicator was created that assessed the presence of EDS and/or insomnia called sleep disorder indicator.

Sleep latency was determined based on the Pittsburgh Sleep Quality Index (PSQI) domains. The PSQI assesses sleep quality in seven domains: subjective quality, sleep latency, sleep duration, sleep efficiency, sleep disorders, use of sleeping medication and dysfunction during the day. The score ranges from 0 to 20, with individuals with a score greater than 5 being considered having poor sleep quality. Snoring was estimated by the Stanford Snoring Scale.^15^

The body mass index (BMI) was measured in all participants, based on the individual’s height and mass. Weight was obtained using a GEOM™ scale, model B8030, with a capacity of 150 kg and sensitivity of 100 g. Height was measured using a Wiso® adult compact measure tape stadiometer graded in centimeters. If the participant was unable to walk to the scale or adopt the orthostatic posture to have the height measured, they were excluded from the study. In addition, abdominal circumference (AC) data were collected using an inelastic measuring tape, divided into low and high cardiovascular risk participants, with the following references as the cutoff point: <84 cm for women and <104 cm for men.^16^ A dichotomous nutritional index was constructed as adequate (BMI <27 kg/m^2^ and/or low cardiovascular risk) and inadequate (BMI ≥27 kg/m^2^ and/or high cardiovascular risk).

To assess the existence of depressive symptoms in the elderly, the Sheikh and Yesavage^17^ Geriatric Depression Scale was used, where scores from 0 to 10 are equivalent to normal status, 11 to 20 refer to mild depressive conditions and 21 to 30 points indicate possible moderate/severe depression.

The researchers were adequately trained to use the data collection instruments reliably before applying the socioeconomic questionnaire, collecting anthropometry data, applying the Geriatric Depression Scale, Epworth Sleepiness Scale and the PSQI.

The second wave of the survey was carried out in 2017. The researchers used the telephone contact strategy initially, followed by home visits, visits to the Basic Health Unit and contact with key informants, such as neighbors and relatives to locate participants and outcomes. After the participants were located, in order to estimate the outcomes, the participant’s vital state (alive or dead) and the occurrence of cardiovascular and cerebrovascular disorders were verified. The events of acute myocardial infarction, arrhythmias, valvulopathies, transient ischemic attack, ischemic or hemorrhagic stroke, among others, were self-reported by the participants or their family members and confirmed from/added to the medical records in the health units and or death certificates. for those already deceased.

### Data analysis

The inferential analysis was performed with death as the primary outcome and cardio-cerebrovascular events as the secondary one, controlling the effect of covariates such as hypertension and diabetes. The relative risk for the independent variables of the EDS/insomnia group was estimated, as well as depressive symptoms, sleep quality parameters and obesity using the Generalized Linear Modeling with a model based on Poisson distribution and the linear logarithmic function. In this inferential situation, a 5% significance level was adopted to minimize type I error. The free software R was used to carry out the analysis of descriptive and inferential data.

The research was submitted to the Research Ethics Committee of Hospital Universitário Onofre Lopes, in compliance with Resolution 466/2012 of the National Health Council, obtaining the approval number 2,048,708.

## Results

The 160 elderly participants were aged between 60-98 years, with a mean age of 72.16 ± 7.84 years; 112 (71.2%) were women and 135 (84.4%) had complete elementary school as their highest level of schooling. The average follow-up time was 5.3 years. There were only four follow-up losses.

In this sample, 118 individuals (73.75%: 66.93-80.56) had normal results regarding EDS; 30 (18.75%: 12.70-24.79) had a mild degree of EDS and 12 (7.50 %: 3.41-11.58) had the severe form of EDS. Insomnia was present in 98 (54.9%) of the elderly, 65 (43.0%) of which at the moderate / severe stage. The outcome death was recorded for 40 elderly people (25.97%: 19.04-32.89) and the outcome cardiovascular events occurred in 48 of them (30.76%: 23.52-38.01). Table 1 describes the distribution of the other independent variables as well as the predictive modeling.

**Table 1:** – Descriptive analysis, unadjusted and adjusted model for the risk of death in the elderly in the municipality of Campina Grande, Paraíba, Brazil, 2009-2017

	Risk of death				
Variables	Unadjusted Model			Adjusted Model	
	n (%)	RR	95%IC	RR	95% IC
**Gender**					
Male	44 (28.2)	1.69	1.01-2.87	1.73	0.92-3.28
Female	112 (71.8)	1		1	
**Marital Status**					
With partner	37 (23.7)	1.59	0.91-2.77	1.50	0.86-2.62
Without partner	119 (76.3)	1		1	
**Depression**					
Yes	66 (42.3)	1.66	0.97-2.84	2.39	1.52-4.56
No	90 (57.7)	1		1	
**Nutritional Index**					
Inappropriate	36 (23.1)	0.76	0.42-1.38	1.12	0.58-2.18
Adequate	120 (76.9)	1		1	
**Hypertension/Diabetes**					
Yes	114 (73.1)	0.86	0.48-1.53	0.84	0.46-1.51
No	42 (26.9)	1		1	
**Sleep Disorder Indicator**					
Insomnia /EDS	114 (81.3)	0.91	0.40-2.07	0.62	0.33-1.16
Normal	46 (28.7)	1		1	
**PSQI**					
Good	62 (45.9)	0.78	0.43-1.41	0.62	0.33-1.16
Bad	73 (54.1)	1		1	

*95%CI: 95% confidence interval; RR: relative risk; EDS: excessive daytime sleepiness; PSQI: Pittsburg Sleep Quality Index.*

The Poisson modeling revealed, after adjustment, that among the studied independent variables, only the presence of depressive symptoms is considered a risk factor for death in elderly community members. Elderly people with depression were 2.39 times (95%CI: 1.52-4.56) more likely to die than those without depressive symptoms. On the other hand, the composite indicators of nutritional status, sleep disorders, sleep quality and even the presence of hypertension/diabetes were not able to demonstrate any influence on mortality during the eight-year follow-up of the study ( Table 2 ).

**Table 2:** – Adjusted model segregated in specific sleep morbidities for the risk of death in the elderly in the city of Campina Grande, Paraíba, Brazil, 2009-2017

	Risk of death		
Variables		Adjusted Model	
	n (%)	RR	95% CI
**Gender**			
Male	44 (28.2)	1.88	1.01-3.50
Female	112 (71.8)	1	
**Marital Status**			
With partner	37 (23.7)	2.10	1.20-3.68
Without partner	119 (76.3)	1	
**Depression**			
Yes	66 (42.3)	2.04	1.06-389
No	90 (57.7)	1	
**Hypertension/Diabetes**			
Yes	114 (73.1)	1.19	0.54-2.60
No	42 (26.9)	1	
**Nutritional Index**			
Inappropriate	36 (23.1)	1.27	0.59-2.71
Adequate	120 (76.9)	1	
**Sleep Latency**			
60 min or more	22 (16.2)	1.68	0.74-3.81
31-60 min	19 (14.0)	3.54	1.26-9.94
16-30 min	31 (22.8)	2.23	1.12-4.47
Less than 15 min	64 (47.1)	1	
**Severity of Insomnia**			
Moderate/Severe	65 (43.0)	0.94	0.29-2.96
Mild	33 (21.9)	2.30	1.08-4.89
Without insomnia	53 (35.1)	1	
**Snoring severity**			
Excessive	38 (26.2)	0.69	0.31-1.53
Mild	54 (37.2)	1.24	0,58-2,65
Without Snoring	53 (36.6)	1	
**EDS**			
Moderate/Severe	12 (7.7)	1.08	0.43-2.68
Mild	29 (18.6)	0.60	0.24-1.53
Without EDS	115 (73.7)	1	

*95%CI: 95% confidence interval; RR: relative risk; EDS: excessive daytime sleepiness.*

When analyzing the variables that make up the composite indicators included in the previous model, a new distribution for the death outcome was identified ( Table 3 ). Having depressive symptoms remained a risk factor for death. However, it is clear that the prediction of death is higher in males, 88% more than in women (RR = 1.88; 1.01-3.50), and also in the elderly with a partner (RR=2.10; 1.20-3.68). Similarly, sleep latency indicators between 16-30 minutes (RR=2.23; 1.12-4.47) and between 31-60 minutes (RR=3.54; 1.26-9.94) predict more deaths than those who fall asleep in up to 15 minutes. Having mild insomnia was also considered a predictor of death in this sample, and those with a mild degree have 2.30 (95%CI: 1.08-4.89) more chances of dying.

**Table 3:** – Predictive model for the risk of cardio-cerebrovascular event in the elderly in the city of Campina Grande, Paraíba, Brazil, 2009-2017

Variables	Risk of CC			
	Unadjusted Model		Adjusted Model	
	RR	95% CI	RR	95% CI
**Gender**				
Male	1.19	0.72-1.98	1.23	0.69-2.21
Female	1		1	
**Marital Status**				
With partner	0.98	0.55-1.72	1,03	0.58-1.82
Without partner	1		1	
**Depression**				
Yes	1.14	0.71-1.83	1,29	0.78-2.15
No	1		1	
**Nutritional Index**				
Inappropriate	0.64	0.33-1.25	0,94	0.55-1.62
Adequate	1		1	
**Hypertension /Diabetes**				
Yes	4.43	1.69-11.64	5,72	1.87-17.46
No	1		1	
**Sleep Disorder Indicator**				
Yes	0.72	0.32-1.63	0,66	0.31-1.38
No	1		1	
**PSQI**				
Good	1.27	0.76-2.10	1,36	0.82-2.27
Bad	1		1	

*CC: Cardio-Cerebrovascular Events; 95%CI: 95% confidence interval; RR: relative risk; EDS: excessive daytime sleepiness; PSQI: Pittsburg Sleep Quality Index.*

For the outcome of a cardio-cerebrovascular event in the eight-year follow-up, the only independent variable that showed a predictive capacity for this outcome was the hypertension / diabetes indicator. Hypertensive and / or diabetic elderly people were almost six times more likely (RR=5.72; 95%CI: 1.87-17.46) to have cerebral cardiovascular disorders than normotensive elderly individuals and those with normal glycemic metabolism. The composite and segregated indicators of nutritional status, sleep disturbance and sleep quality did not reveal any effect in the analyzed period ( Table 4 ).

**Table 4:** – Predictive model segregated in specific sleep morbidities for the risk of cardio-cerebrovascular events in the elderly in the city of Campina Grande, Paraíba, Brazil, 2009-2017

Variables	Risk of CC	
	Adjusted Model	
	RR	95%CI
**Gender**		
Male	1.38	0.59-3.21
Female	1	
**Marital Status**		
With partner	1.32	0.62-2.82
Without partner	1	
**Depression**		
Yes	1.32	0.67-2.59
No	1	
**Hypertension/Diabetes**		
Yes	8.30	1.98-34.82
No	1	
**Nutritional Index**		
Inappropriate	1.18	0.58-2.41
Adequate	1	
**Sleep Latency**		
60 min or more	1.03	0.40-2.62
31-60 min	1.34	0.50-3.55
16-30 min	1.09	0.45-2.59
Less than 15 min	1	
**Severity of Insomnia**		
Moderate/Severe	1.35	0.55-3.33
Mild	2.14	0.84-5.42
Without insomnia	1	
**Snoring severity**		
Excessive	0.99	0.41-2.41
Mild	0.96	0.44-2.07
Without Snoring	1	
**EDS**		
Moderate/Severe	0.79	0.26-2.43
Mild	0.74	0.28-1.97
Without EDS	1	

*CC: Cardio-Cerebrovascular Events; 95%CI: 95% confidence interval; RR: relative risk; EDS: excessive daytime sleepiness.*

## Discussion

It is evident in the present study that the assessed outcomes of death and cardiovascular events are predicted by different exposures. The death of elderly community members is strongly influenced by the occurrence of depressive symptoms, with death being more common in men and in individuals living with a partner. Among the sleep characteristics, the high latency to initiate sleep and the severity of insomnia stand out as risk factors for death in the elderly. On the other hand, only hypertensive and/or diabetic elderly people showed a higher risk of developing cardiovascular outcomes.

Several studies have clarified the higher risk of death among men, mainly due to chronic conditions such as cardiovascular diseases in the elderly.^18^ Marital status is also identified as a condition related to health vulnerability, since individuals without partners usually have less social support and greater predisposition to harmful health events, such as death.^19^ However, our findings suggest that elderly people with a partner are more vulnerable to death. The suggested explanatory hypothesis is based on the characteristics of the studied population, where the possibility of interaction between low socioeconomic conditions and low social support overburdening the spouses with reciprocal or vulnerable care. Data from the National Health Survey also point to a greater occurrence of a decline in sleep repair in individuals with a partner in Brazil.

It was shown that depressive symptoms in the elderly are risk factors for death. Depressive symptoms predicted death events for all causes, showing an interaction with the male gender.^20^ It was also identified that depressive symptoms are predictive of mortality from cardiac ischemia in a cohort of the general population in England,^21^ which corroborates evidence that indicates depression as the main chronic morbidity that currently affects the elderly^22^ and that may have a direct effect or interaction with other pathological mechanisms.

Depressive conditions are usually related to sleep disorders. Lima at al.^23^ reported that elderly women with fewer hours of sleep had worse mental health indicators regarding the emotional aspects in a population study in Brazil.^23^ Symptoms of insomnia and drowsiness are predictive of depression. It is possible that these conditions establish a feedback cycle that culminates in the death outcome.^3^

Depression as a predictor of death in the elderly is related to the worsening of chronic comorbidities.^24^ Depression in the elderly, when associated with chronic morbidities, makes it difficult to manage other conditions, such as diabetes and hypertension, for example, especially regarding adherence to treatment and self-care.^25^ Additionally, it also changes both pain^26^ and general health status^4^ perception, creating clinical manifestations that are oversized in relation to the real pathophysiological problem.

Our findings point to an exposure to the risk of death produced by increased sleep latency and the severity of insomnia in the elderly. Similarly, Lallukka et al.^3^ revealed that in Norway and Finland, insomnia increases the likelihood of death in men,^3^ as well as observed in the United States,^27^ mainly due to their difficulty in initiating sleep. Insomnia is strongly associated with other factors that predict death, such as reduced or elevated sleep duration^28^ and depression in the elderly. On the other hand, Chen et al.^29^ identified independence between insomnia and death events in elderly Asian people in nine years of follow-up.^29^ Such discrepancies may be due to contextual differences or insufficient time of follow-up for the outcome to occur.

Unlike other studies, the composite indicator of excessive daytime sleepiness and insomnia complaints, as well as its single indicators, are not predictive factors for cardiovascular events. However, Wu et al.^2^ showed in a cohort from Taiwan that insomnia increases the risk of cerebrovascular event, especially in young adults compared to the elderly. Cardiac events were also predicted by the presence of insomnia symptoms in other epidemiological findings.^10 , 30^

As for the outcome of cardiovascular events, our data do not indicate sleep or emotional conditions as risk factors. The only identified health conditions that were risk predictors were hypertension and / or diabetes in the elderly in the analyzed period. The scientific literature had already shown, with enough support, the deleterious effects that systemic arterial hypertension and diabetes have on the contemporary society, being a robust risk factor or component for ischemic^31^ and hemorrhagic cerebrovascular events, cardiac ischemia and metabolic syndrome.^32^

Some points deserve to be highlighted when interpreting our findings. Despite the methodological rigor in the follow-up planning, important social variables such as income, social support network or social status were not controlled, which would well reflect the contextual aspects involved in exposures and outcomes. The low level of schooling in the studied population did not allow the observation of good variability regarding this characteristic and, therefore, greater inferences about its effects. The lack of good health knowledge and/or access to health services in the sample prevented a more accurate identification of possible subclinical cardiovascular events, which were identified by self-report. The lack of a diagnostic test for sleep apnea made it impossible to control the effect of this health condition on its likely interaction with sleep characteristics. Finally, the adjustment of covariates was limited by the sample size.

However, important information was obtained for the assessed elderly population, located on the peripheries of large urban centers in Brazil, in a region with peculiar sociodemographic and economic characteristics, and with few follow-up studies for vulnerable subpopulations. Thus, the identification of the independent effects that sleep and depressive symptoms have on mortality in the elderly are of considerable importance for health policies aimed at the elderly, especially for public and clinical planning. This information will be useful to support decision-making regarding the organization of care for the elderly, mainly to mitigate the predisposing vulnerabilities related to their functional capacity and survival.

## Conclusion

We identified in the assessed sample independent effects that depressive symptoms, sleep latency and insomnia severity have on the mortality of elderly people. Such conditions are characterized by their modifiable capacity and, therefore, the possibility of minimizing the probability of death if they are counteracted by appropriate management and at the adequate level of care. There are also the effects related to the issue of the male gender being more prone to death. This refers to the need of sectoral strategies related to both health services access and work conditions, as well as social assistance to resolve inequities.

As for the cardio-cerebrovascular events, only the presence of hypertension and/or diabetes increased their risk in elderly community members. Although they are well-known risk factors, there are gaps in the appropriate management of these conditions when it comes to adherence to treatment and access to health services and goods, which are influenced by social issues related to income, level of schooling and gender, aiming to achieve better results in public health.
